# Country and Sex Differences in Decision Making Under Uncertainty and Risk

**DOI:** 10.3389/fpsyg.2020.00486

**Published:** 2020-03-20

**Authors:** Varsha Singh, Johannes Schiebener, Silke M. Müller, Magnus Liebherr, Matthias Brand, Melissa T. Buelow

**Affiliations:** ^1^Department of Humanities and Social Sciences, Indian Institute of Technology Delhi, New Delhi, India; ^2^Department of General Psychology Cognition, University of Duisburg-Essen, Duisburg, Germany; ^3^Department of Psychology, The Ohio State University, Newark, OH, United States

**Keywords:** sex differences, gender, iowa gambling task, decision making, risk, uncertainty

## Abstract

Whether males and females differ in decision-making remains highly debatable. However, a male advantage in decision making is observed in animal as well as human models of the iowa gambling task (IGT), and, in case of the latter, the difference is observed across a wide range of age groups. It is unclear if these sex differences on the IGT are malleable to environmental influences such as sociocultural factors. We tested sex differences during the uncertainty and risk phases of the IGT in data pooled from three countries that reflected high, moderate, to low gender-equity (Germany, United States, and India: *N* = 531, female = 269). Comparing the net scores in uncertainty vs. risk blocks (first two vs. last two blocks) confirmed the male-advantage on the IGT across the three countries, specifically in the risk blocks, with the highest male-advantage observed for Germany. Results are discussed in terms of sex differences in reaction to uncertainty vs. risk, and the counter-intuitive effect of gender-equitable environment suggesting that national/environmental factors might influence advantageous decision making, but in ways that accentuate rather than abate sex differences.

## Introduction

Sex differences on the iowa gambling task (IGT) are well-documented in human and animal models, suggesting sex-specific decision making processes (van den Bos et al., 2012, 2013). Further, the female decision-making deficit is prominent in the early task phase that is associated with uncertainty/ambiguity (Bolla et al., 2004) while the male-advantage is characterized by higher advantageous decision making in the last phase of the task, when the decision payoffs are known (i.e., decision making under risk; van den Bos et al., 2013). The authors of the IGT proposed neurobiological differences in anatomy of the prefrontal cortex (PFC), specifically right-PFC engagement in males (Tranel et al., 2005) and left-dorsolateral PFC engagement in females (Bolla et al., 2004) contributing to sex differences on the task. However, age-related improvements due to PFC-maturation is reported for both males and females, specifically in the last phase of the task (Crone and van der Molen, 2004; Hooper et al., 2004). It remains unknown whether sex-differences on the IGT, specifically phase-specific male-advantage in advantageous decision making, will be observed in countries that differ in sociocultural environment.

In spite of efforts toward cultural adaptation of the task (e.g., Rutz et al., 2013), potential sex-differences in phase-specific IGT performance across countries that differ in sociocultural and gender-equitable environment remain unexplored. For instance, decision making in the risk phase of IGT differed between two culturally different countries but showed no effect of age or gender (e.g., Brazil and United States) (Bakos et al., 2010). Compared to American participants, Israeli participants performed poorly on the IGT, and the authors pointed out that country-differences might become prominent when American vs. non-American population is compared; however, in the absence of the sex composition of the Israeli sample and phase-specific analysis of IGT, it is unclear whether between-country differences in gender-equity and socioeconomic status between America and Israel influenced sex- and phase-specific decision making (Ekhtiari et al., 2009). Our previous work (within/single-country analysis) pointed toward phase-specific sex-differences in the IGT. For instance, IGT performance declined after the uncertainty blocks for strongly right-handed Indian females (Singh, 2016), presence of female-dominant sample in the United States was linked with poor deck choice (deck B) in the uncertainty phase of the IGT (Okdie et al., 2016), and stress-induced IGT deficit was high in German males whereas the non-stressed males continued to show male-advantage in the task (Starcke et al., 2017). Even though the IGT is a non-linguistic measure of neuropsychological assessment, authors have cautioned that country and cultural differences should be considered in task interpretation (Fasfous et al., 2013; Daugherty et al., 2017). These observations, combined with reports of sociocultural factors potentially influencing the IGT (Ekhtiari et al., 2009; Bakos et al., 2010), or cultural variation in the IGT as a part of neuropsychological assessment (Fasfous et al., 2013; Daugherty et al., 2017), prompted us to analyze potential country and sex interactions in two distinct phases of IGT decision making (i.e., uncertainty and risk phases).

Sex and country-based comparisons of performance on widely used cognitive tasks of risk and decision making might reflect the effect of socioeconomic environment, such as gender-equity, on task performance, and additionally might explain societal outcomes such as female underrepresentation in fields that are cognition-intensive, working memory dependent, and male-dominated, such as math (Reilly, 2012). Further, decision making in the last phase of the IGT implicates PFC-governed executive functions (Brand et al., 2007), whereas the first two blocks of the uncertainty phase are least affected by working memory demands (Bagneux et al., 2013). Less is known about male advantage and country-level variation in executive functions; however, working memory shows a male-advantage that is independent of ethnicity (Silverman et al., 2007) and gender-related attitude (Lippa et al., 2010). On the other hand, country-variation in gender equity influenced sex differences in working memory (Miller and Halpern, 2014). We selected three countries that reflect a gradation in gender-equity – Germany representing the high gender-equitable country, United States representing moderate gender-equity, and India representing the low gender equitable country (Germany ranked 10th, United States ranked 53rd, and Indian ranked 112th, World Economic Forum, 2019). Therefore, the aim of the present study was to investigate whether sex differences on the IGT are phase and/or country-specific.

## Materials and Methods

Data were pooled from unpublished datasets from three countries: India, United States, and Germany. The sample (*N* = 531) consisted of 269 (50.7%) female and 262 (49.3%) male participants between 18 and 35 years of age (*M* = 22.62, SD = 3.74). The Indian sample was collected from the city of New Delhi (*n* = 177, 85 females) and contained participants with a maximum age of 35 years (*M* = 23.31, SD = 2.99). From originally 184 subjects from Germany (city of Duisburg) and 743 subjects from the United States (city of Newark, OH), one sub-sample each was selected that matched the sample from India in terms of size (i.e., *n* = 177), age distribution (with a maximum of 35 years), and gender distribution (i.e., similar number of males and females). The selected sample from Germany (*n* = 177) contained 96 females and 81 males aged between 18 and 33 years (*M* = 24.25, SD = 3.84). The selected sample from the United States consisted of 177 participants (88 females) between 18 and 34 years (*M* = 20.29, SD = 3.13). The United States data were skewed in terms of age and gender distribution. Therefore, participants were selected to represent age (median-based groups) and sex in the following manner. First, an all-male United States sample was created taking all male participants ages 20–35 years (21 cases), then 51 male 19-year-old participants, then 17 male 18-year-old participants. The same procedure was used to select the 88 female participants.

The data from each country were collected as a part of research protocols approved by institutional ethics committees where the studies were conducted. Informed consent of the participants for research participation and for publication of its results was collected as a part of the protocols. Participants had more than 12 years of formal education (post-secondary level), and were students enrolled in an education program in the public/national institute where the studies were carried out. We assumed that the three countries reflect the place of education of the participant, and of data collection, rather than a measure of cultural and national identity of the participant. Information regarding age, gender, and block-wise net score was pooled from the three datasets. The datasets from the three countries did not differ regarding gender distribution [χ^2^(2) = 1.46, *p* = 0.482], but regarding age [*F*(2,531) = 67.86, *p* < 0.001, $\eta_{\text{p}}^{2}=0.204$], which is why we controlled for age in all of the following analyses. The IGT performance was represented by block-wise (i.e., five blocks of 20 trials each) net scores as per the standard scoring approach: number of cards drawn from decks C′ and D′ minus the number of cards drawn from decks A′ and B′. Additionally, net scores on blocks 1 and 2 (trials 1–40) were totaled to reflect decision making under uncertainty (early phase) and net scores on blocks 4 and 5 (trials 61–100) were totaled to reflect decision making under risk (late phase).

### Measures

Computerized version of the IGT was used with progressive reward variant (A′, B′, C′, D′) and standard task instructions in the respective local language. Task performance was non-incentivized for all the participants (there were no performance-contingent incentive provided to the participants).

### Analysis

A mixed model ANOVA was used to first address net scores for task progression across the five blocks of trials, and then to address phase-specific net scores (blocks: uncertainty vs. risk) as the within-subject variable and gender (male vs. female), and country (India vs. United States vs. Germany) as between-subject variables with age as a covariate.

## Results

**FIGURE 1:**
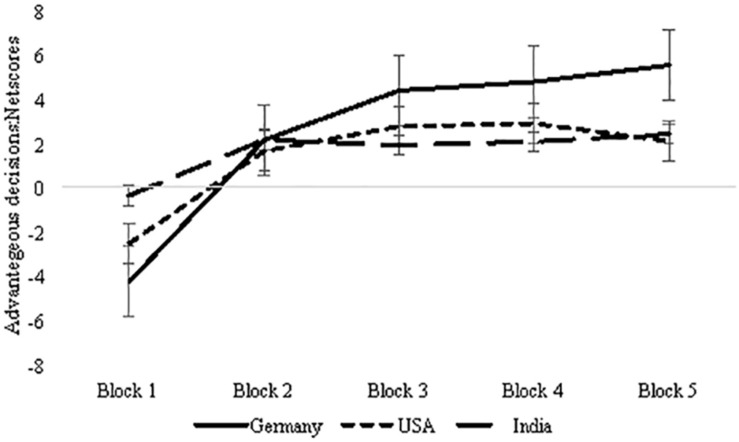
Three-country comparison of advantageous decision making as the task progresses over 100 trials. Error bars represent standard error.

**FIGURE 2:**
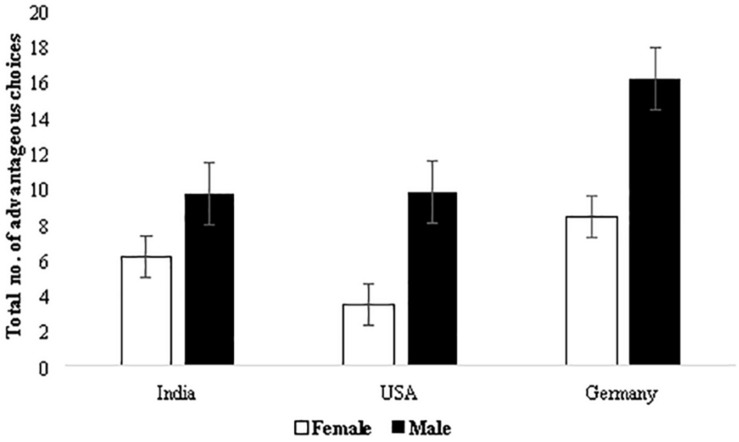
Country and sex-specific comparison of advantageous decision making in 100 trials of the IGT. Error bars represent standard error.

Results revealed a main effect of the five blocks suggesting that advantageous decision making improved as the task progressed, *F*(3.47, 1816.87) = 3.73, *p* < 0.01, $\eta_{\text{p}}^{2}=0.007$ (mean net score: block 1 = −2.45, block 2 = 1.92, block 3 = 2.96, block 4 = 3.19, and block 5 = 3.29) (Greenhouse-Geisser corrected values, Table 1). The interaction of sex and block was not significant, *F*(3.47, 1816.87) = 1.50, *p* = 0.21, $\eta_{\text{p}}^{2}=0.003$, and neither was that of age and block, *F*(3.47, 1816.87) = 1.37, *p* = 0.243, $\eta_{\text{p}}^{2}=0.003$. Task progression and improvement in long-term decision making was observed independent of sex and age. As expected, the interaction of country and block was significant, *F*(6.93, 1816.87) = 8.75, *p* < 0.001, $\eta_{\text{p}}^{2}=0.032$, suggesting that participants from the three countries differed in advantageous decisions made as the task progressed with participants from Germany making the most advantageous decisions (Figure 1) (mean net scores: Germany = 2.51, India = 1.61, and United States = 1.23). The three-way interaction of block, country, and sex was significant, *F*(6.94, 1800.19) = 2.24, *p* = 0.029, $\eta_{\text{p}}^{2}=0.009$, suggesting that both sex and country had a small influence on improvement in advantageous decision making (Figure 2) (mean net scores: Germany male = 3.27 vs. female = 1.74, United States male = 1.94 vs. female = 0.53, and India male = 1.97 vs. female = 1.24), with the greatest difference between the net scores of males and females for Germany.

**TABLE 1:** Summary of the mixed ANOVA including the factors IGT block (within), sex (between), and country (between) on IGT net score.

**Main effects and interaction effects**	***F***	***p***	**$\eta_{\text{p}}^{2}$**
Block (five levels)^∗^	3.73	0.008	0.007
Sex (two levels)	5.25	0.022	0.010
Country (three levels)	1.71	0.182	0.006
Block × sex	1.50	0.206	0.003
Block × country	8.75	<0.001	0.032
Block × sex × country*	2.24	0.029	0.009

IGT, iowa gambling task. Effects are controlled for age. * Greenhouse-Geisser corrected.

**FIGURE 3:**
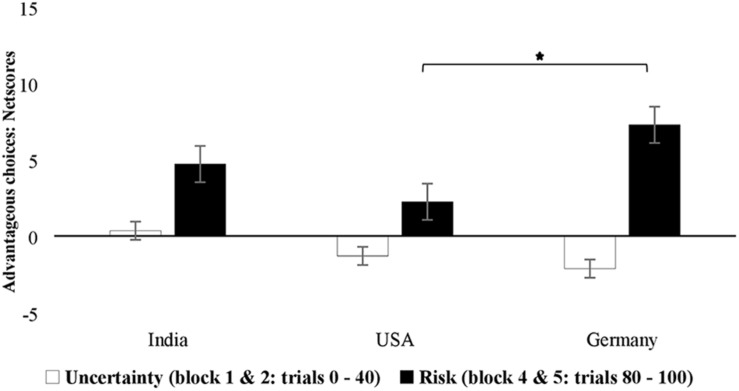
Country-specific advantageous decision making of female participants under uncertainty (trials 0–40) and risk phase of the IGT (trials 80–100). Error bars represent standard error.

**FIGURE 4:**
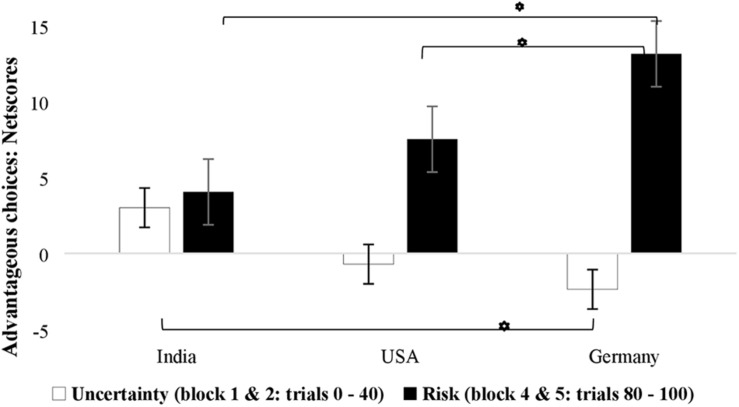
Country-wise advantageous decision making of male participants under uncertainty (trials 0–40) and risk phase of the IGT (trials 80–100) suggests greater change in advantageous decision making from uncertainty to the risk phase occurred in case of Germany. Error bars represent standard error.

Results of decision making in the uncertainty trials (early phase) and risk trials (late phase) showed no main effect of block, *F*(1,524) = 1.93, *p* = 0.166, $\eta_{\text{p}}^{2}=0.004$, and no main effect of country, *F*(2, 524) = 1.16, *p* = 0.313, $\eta_{\text{p}}^{2}=0.004$, but a significant main effect of sex, *F*(1,524) = 4.97, *p* = 0.026, $\eta_{\text{p}}^{2}=0.009$ (Table 2). Looking at the interactions, there was no effect of sex and block, *F*(1,524) = 2.82, *p* = 0.094, $\eta_{\text{p}}^{2}=0.005$. The interaction of age and block was also not significant, *F*(1,524) = 0.002, *p* = 0.962, $\eta_{\text{p}}^{2}=0.001$. The interaction between country and block was significant, *F*(2,524) = 15.76, *p* < 0.001, $\eta_{\text{p}}^{2}=0.057$ (mean net scores: Germany uncertainty blocks = −2.20 vs. risk blocks = 10.24, United States uncertainty blocks = −1.10 vs. risk blocks = 4.81, and India uncertainty blocks = 1.71 vs. risk blocks = 4.39). The three-way interaction between block, sex, and country was significant, *F*(2,524) = 4.18, *p* = 0.016, $\eta_{\text{p}}^{2}=0.016$, suggesting that male and female participants from the three countries differed in advantageous decisions made in the under uncertainty and under risk portions of the task with the highest improvements in decision making from uncertainty to risk phase being observed for Germany (Figures 3, 4).

**TABLE 2:** Summary of the mixed ANOVA including the factors IGT phase (within), sex (between), and country (between) on IGT net score.

**Main effects and interaction effects**	***F***	***p***	**$\eta_{\text{p}}^{2}$**
IGT phase (two levels)*	1.93	0.166	0.004
Sex (two levels)	4.97	0.026	0.009
Country (three levels)	1.16	0.313	0.004
IGT phase × sex	2.82	0.094	0.005
IGT phase × country	15.76	<0.001	0.057
IGT phase × sex × country	4.18	0.016	0.016

*IGT, iowa gambling task. Effects are controlled for age. ^∗^Greenhouse-Geisser corrected.*

In order to analyze the reported three-way interaction in more detail, we ran additional analyses split by sex, country, and block respectively. Age was again included as a covariate. *Post hoc* ANOVA separated by sex showed that the block × country interaction was more pronounced in males [*F*(2, 258) = 15.00, *p* < 0.001, $\eta_{\text{p}}^{2}=0.104$] compared to females, *F*(2, 265) = 3.02, *p* = 0.050, $\eta_{\text{p}}^{2}=0.022$ (see Figures 3, 4 for a visualization). *Post hoc* ANOVA split by country showed the effects of block and sex were significant only in the case of Germany, *F*(1,174) = 7.53, *p* = 0.007, $\eta_{\text{p}}^{2}=0.041$, as these were not significant for United States, *F*(1,174) = 2.58, *p* = 0.110, $\eta_{\text{p}}^{2}=0.015$, or for India, *F*(1,174) = 1.63, *p* = 0.203, $\eta_{\text{p}}^{2}=0.009$. Separate comparisons for the uncertainty and risk phases of the IGT showed no effect of sex in the (early) uncertainty phase, *F*(1, 224) = 1.26, *p* = 0.262, $\eta_{\text{p}}^{2}=0.002$, but an effect in the (later) risk phase, *F*(1, 224) = 5.70, *p* = 0.017, $\eta_{\text{p}}^{2}=0.011$, with males performing better than females, especially in the sample from Germany (see Figures 3, 4).

This three-country comparison was based on participants drawn from large/national educational institutions that are representative of the diverse population of their country. To rule out the possibility that the results might be affected due to non-representativeness of the sub-sample drawn from the United States sample, we carried out the same set of analysis on another age and gender matched sub-sample from the United States sample. We found support for the results obtained in the earlier iteration suggesting consistency in our findings reported herewith, that is, there were country, and sex-differences in phase-specific IGT performance, with high long-term decision-making and high male-advantage observed for Germany. Additionally, to check whether sampling variation was under control, we used retrospective power analysis (G^∗^Power) and confirmed that the values obtained in *F* tests were well within the limits of the critical *F*, and that the beta error (type II error) were within the acceptable limits (Banerjee et al., 2009).

## Discussion

The present investigation aimed to explore sex and country-wise differences in long-term decision making; analysis of the five blocks showed improvement in long-term decision making and, even though the change was independent of sex, the task progression varied across country, showing the highest task improvement for Germany. We observed that advantageous decision-making improved as the task progressed, and this varied by country and the combined effect of country and sex. There was a country-wise difference in advantageous decision making; however, the effect size was small. High net scores in Germany could be because the male advantage in working memory is high in Germany compared to other countries (Janssen and Geiser, 2012; Jansen et al., 2016).

We specifically explored whether the male advantage occurred in the uncertainty phase (early phase) or in the risk phase (later phase) and whether the phase-specific male advantage varied with age and countries with gender-favorable environment. Age had no effect on decision making on the IGT, and its interactions with sex and country also failed to influence decision making. These results are in line with others who observed that the male advantage on the IGT was consistent in adolescents and older adults (Overman and Pierce, 2013). Further, age-related improvement in IGT was unaffected by cultural differences in an 11-country comparison of western and Asian countries (including United States and India) (Icenogle et al., 2017), suggesting that the effect of age and maturation on IGT decision making might be the same across the three countries.

Sex had a significant effect on advantageous decision making on the IGT, beyond the effects of country, age, and IGT phase, illustrating an overall male advantage. Furthermore, sex and country jointly influenced decision making on the IGT and differed during the decision making under uncertainty vs. risk phases of the task. This finding is in line with others who found that advantageous decision making among males and females differed between the under risk and uncertainty trials (Bolla et al., 2004; van den Bos et al., 2013). Further, advantageous decision making differed between uncertainty and the risk phase of the task, and the interaction of country and sex contributed to this difference. Males outperformed females in advantageous decision making and the male advantage was most prominent in the risk-phase of the task. Further, the male advantage during the risk phase was higher in Germany, a country with high gender-equitable environment compared to the other two countries that rank lower in gender-equity (World Economic Forum, 2016). These results are aligned with a counter-intuitive observation that cognitive sex differences are accentuated in nations that have high gender equity and are economically developed (Lippa et al., 2010; Stoet et al., 2016). Others have also found that sex-differences in personality measures tend to be largest in countries that have more gender-equitable environment (Mac Giolla and Kajonius, 2018). Why would sex differences be larger in a country that has macro environment that facilitates gender equity?

Studies have suggested that highly industrialized and gender-equitable countries have higher male-advantage in working memory (Lippa et al., 2010; Janssen and Geiser, 2012; Jansen et al., 2016). It is possible that high male-advantage in working memory contributed to country and sex-differences in phase-specific IGT performance. Further, the prominent male-advantage in Germany could also reflect population-level variation in testosterone, the male sex hormone. Testosterone is unaffected by race/ethnicity (Alvarado, 2010) and is higher in Western or industrialized/developed countries (e.g., United States) (Ellison et al., 2002); however, male testosterone decline is higher in the United States than it is in Germany (Anaissie et al., 2017). Since testosterone drives risk taking in the IGT (Reavis and Overman, 2001) and high male advantage in working memory in Germany is testosterone-linked (Jansen et al., 2019), it is possible that country-level differences in testosterone resulted in accentuated phase-specific sex differences in the IGT performance in Germany.

A second possible explanation for such results might be that female participants from countries that have low gender equity probably worked harder and put more efforts toward reducing the gender-gap to reach the level of higher education. Operating in challenging environment (i.e., low gender equitability) might have altered decision making of these females to match that of the males from these countries. Introducing effortful deliberation through moral dilemmas improved IGT decision making of females to the level of male participants, with additional evidence suggesting this increased deliberation led to engagement of the dorsolateral PFC with subsequent improvement of working memory and advantageous decision making of females (Overman et al., 2006). Since males engage dorsolateral PFC (as compared to the medial orbital frontal cortex engaged by females), the group used an olfactory task to engage the medial orbital frontal cortex and found it to reduce advantageous decision making of males to equal that of females (Overman et al., 2011). Previous studies have shown that the dorsolateral PFC is especially relevant for advantageous decisions under risk (Labudda et al., 2008), and therefore might have played a critical role in the later phases of the IGT that is marked with risk (Brand et al., 2007). In line with the former, the results of the current study showed that the gender differences were more pronounced in the later phase of IGT. Further, everyday stressors in low industrialized and developed countries might influence working memory differentially for males and females as stress-induced impairment is prominent in males (Preston et al., 2007). It might be possible that unfavorable/gender-inequitable environment of low developed countries increases effortful deliberation and working memory in females, whereas stress impairs working memory in males, thereby bridging the gender-gap in working memory in low industrialized and developed countries. By contrast, in countries that have gender equity and are highly industrialized/developed, the gender-gap in working memory remains relatively unchallenged or unchanged.

These results need to be interpreted considering several limitations of this approach, specifically, that the data pooled from three countries did not include explicit measures of cultural or national identity of the participants. Further, we use the term sex and gender interchangeably because we did not account for sexual identity; instead, we relied on binary categories of self-reported sex/gender to reflect female/woman and male/man. The absence of other measures such as mood, personality, and intelligence, and working memory in particular, that can influence task decision making pose a limitation. Similar to other multi-laboratory collaborations that have pooled the IGT data (e.g., Steingroever et al., 2015), the present study lacks information on socioeconomic level, ethnicity/race for the data sets. One redeeming factor might be that the three institutions are public/national institutions and admit students on the basis of performance in a centralized/national-level entrance exam, and drawing students who represent diversity of the national population. All participants from the three countries had completed post-secondary education; however, we did not analyze the effect of years of formal education on the task performance, it is possible that effect of education are sex-specific. For example, a study conducted in Canada found formal education improved decision making in the IGT independent of sex, specifically in the risk trials (blocks 3 and 4 in Figure 1; Davis et al., 2008), however, another study conducted in the United Kingdom observed that formal education had a detrimental effect on IGT performance in an all-female sample, particularly females with less formal education performed well in the last trials assessing risk-taking (Evans et al., 2004). Future efforts should be directed toward building a multi-laboratory data repository of the IGT and similar decision-making tasks, demographic and education-level information, assessment of mood, intelligence, working memory, executive function, and disposition measures of personality, risk taking, and reward sensitivity across diverse population.

Sex differences in reference to cognitive tasks are changing. Our findings (a) confirm the male-advantage in the IGT across three countries; (b) demonstrate the male-advantage in the shift from the uncertainty to the risk phase is common across the three countries, and (c) present insight that the highest male-advantage in uncertainty-risk shift occurred in a sample from the most gender-equitable country. Because cognitive sex differences are gaining importance in neuroscience (Cahill, 2006), future studies should include measures of brain asymmetry and sex-related neurobiological measures such as cortisol and testosterone and test the interaction of structural and neurobiological factors with country/culture-specific factors to determine their contribution to sex-differences in decision making in uncertainty and risk.

So far, the extant literature has explored age, sex, country, and IGT phase-specific differences in isolation, therefore the present results offer insight into the link between sex, age, and country and phase-specific decision-making. Advantageous decision making differed under uncertainty and risk such that advantageous decision making was lowest under uncertainty and highest under risk. In addition, the difference in advantageous decision making based on IGT phase across age, sex, and country. Even though sex and country influenced advantageous decision making, the results suggest that national/environmental factors in the form of country-level variation might additionally influence advantageous decision making, but in ways that counter-intuitively accentuate, rather than abate sex differences.

## Data Availability Statement

The datasets generated for this study are available on request to the corresponding author.

## Ethics Statement

The studies involving human participants were reviewed and approved by an Institute Ethics Committee (India: IIT Delhi; Germany: University of Duisburg-Essen; United States: The Ohio State University, Newark). The participants provided their written informed consent to participate in this study.

## Author Contributions

VS, MTB, SM, and ML: equal contribution to the analysis and writing of the manuscript. JS and MB: initializing/facilitating collaboration. All authors made equal contribution.

## Conflict of Interest

The authors declare that the research was conducted in the absence of any commercial or financial relationships that could be construed as a potential conflict of interest.
